# Leptin Mediates *In Vivo* Neutrophil Migration: Involvement of Tumor Necrosis Factor-Alpha and CXCL1

**DOI:** 10.3389/fimmu.2018.00111

**Published:** 2018-02-06

**Authors:** Glaucia Souza-Almeida, Heloisa D’Avila, Patricia E. Almeida, Tatiana Luna-Gomes, Sally Liechocki, Barbara Walzog, Ingrid Hepper, Hugo Caire Castro-Faria-Neto, Patricia T. Bozza, Christianne Bandeira-Melo, Clarissa M. Maya-Monteiro

**Affiliations:** ^1^Laboratório de Imunofarmacologia, Instituto Oswaldo Cruz, Fundação Oswaldo Cruz, Rio de Janeiro, Brazil; ^2^Instituto de Ciências Biológicas, Universidade Federal de Juiz de Fora, Juiz de Fora, Brazil; ^3^Departamento de Ciências da Natureza, Instituto de Aplicação Fernando Rodrigues da Silveira, Universidade do Estado do Rio de Janeiro, Rio de Janeiro, Brazil; ^4^Walter Brendel Centre of Experimental Medicine, Department of Cardiovascular Physiology and Pathophysiology, Biomedical Center, Ludwig-Maximilians-Universität München, Munich, Germany; ^5^Instituto de Biofisica Carlos Chagas Filho, Universidade Federal do Rio de Janeiro, Rio de Janeiro, Brazil

**Keywords:** leptin, neutrophil, inflammation, tumor necrosis factor-alpha, CXCL1, leukotriene B4

## Abstract

Leptin directly activates macrophages and lymphocytes, but the role of leptin in neutrophil activation and migration is still controversial. Here, we investigate the *in vivo* mechanisms of neutrophil migration induced by leptin. The intraperitoneal injection of leptin (1 mg/kg) induces a time- and concentration-dependent neutrophil influx. We did not observe the enhancement of lipid bodies/droplets in neutrophils, after leptin treatment, as we had observed previously in peritoneal macrophages. The participation of leukotriene B_4_ (LTB_4_) in neutrophil recruitment triggered by leptin was investigated using different strategies. Leptin-induced neutrophil recruitment occurs both in the absence of 5-lipoxygenase activity in 5-lipoxygenase (5-LO)^−/−^ mice and after the administration of either 5-LO inhibitor (Zileuton) or the LTB_4_ receptor antagonist (U-75302). Moreover, no direct induction of LTB_4_ by leptin could be observed. Neutrophil influx could not be prevented by the mammalian target of rapamycin (mTOR) inhibitor, rapamycin, contrasting with the leptin-induced signaling for lipid body formation in macrophage that is mTOR-dependent. Leptin administration led to tumor necrosis factor-alpha (TNFα) production by the peritoneal cells both *in vivo* and *in vitro*. In addition, neutrophil recruitment was inhibited in tumor necrosis factor receptor 1 (TNFR1^−/−^) mice, indicating a role for TNF in leptin-induced neutrophil recruitment to the peritoneal cavity. Leptin-induced neutrophil influx was PI3Kγ-dependent, as it was absent in PI3Kγ^−/−^ mice. Accordingly, leptin induced the peritoneal cells to produce CXCL1, both *in vivo* and *in vitro*, and the neutrophil influx was ablated after using an antibody against CXCL1. Our results establish TNFα/TNFR1- and CXCL1-dependent signaling as important pathways for leptin-induced neutrophil migration *in vivo*.

## Introduction

A growing body of evidence indicates that leptin is a key modulator of the homeostasis of the immune system (1, 2). Although effective leptin therapy for treating obesity and diabetes is not established, mainly due to leptin resistance in obese patients, there are several clinical trials for combined therapies to improve the treatment of obesity-related conditions and lipodystrophy (3–6). In fact, the leptin analog Metreleptin was approved by the U.S. Food and Drug Administration for the long-term treatment of lipodystrophy (3, 7). Leptin is known as an adipokine and modulator of the immune system (8). However, the mechanism of leptin-induced leukocyte activation and recruitment *in vivo* needs further investigation. Leptin has been shown to act as a direct activator of macrophages, lymphocytes, and other leukocytes (9). Neutrophils, however, do not present the full-length leptin receptor, LepRb. This isoform is responsible for the described intracellular effects of leptin in different cell types. Leptin indirectly induces the *in vitro* neutrophil expression of CD11b in response to the direct stimulation of macrophages (10). When investigating the *in vivo* effects of leptin on peritoneal macrophages and lipid droplet formation, we detected the presence of neutrophils in the peritoneal lavage (11). Leptin is known to be important for a proper response to infections and immunological homeostasis, but little is known about the *in vivo* modulation of neutrophil migration by leptin (12). Leptin is acutely enhanced in infections and chronically enhanced during obesity, and neutrophils play an important role on the innate immune response (13, 14). We decided therefore to investigate how leptin can activate and induce inflammatory mediators indirectly causing neutrophil migration *in vivo*, with potential implications for a number of inflammatory conditions in different diseases. TNFα was described to act on the priming step of neutrophil activation, while chemokines as well as leukotriene B_4_ (LTB_4_) can induce neutrophil adhesion and migration (15). TNFα was also found contributing to neutrophil recruitment stimulated by the chemokine CXCL1/KC, a direct chemoattractant for neutrophils (16). Here, we show that exogenous leptin induces *in vivo* migration and persistent neutrophil accumulation in the peritoneal cavity, through a mechanism largely dependent on TNFα and CXCL1 but independent of LTB_4_ production and signaling.

## Materials and Methods

### Materials

Murine-recombinant leptin, rabbit anti-mouse CXCL1 antibody (catalog # 250-11), and control rabbit IgG (catalog # 500-P00) were purchased from Peprotech, Inc. (Rocky Hill, NJ, USA). Rapamycin was obtained from Sigma-Aldrich, Inc. (Saint Louis, MO, USA). Zileuton was obtained from Santa Cruz Biotechnology, Inc. (Dallas, TX, USA). U-75302 was purchased from Cayman Chemical (Ann Arbor, MI, USA). Osmium tetroxide was provided from Ted Pella, Inc. (Redding, CA, USA).

### Animals

We used male mice of different strains: C57Bl/6, C3H/HeJ, C3H/He, 5-lipoxygenase (5-LO)-deficient (5-LO^−/−^), CCL3-deficient (CCL3^−/−^), tumor necrosis factor receptor 1 (TNFR1-deficient) (TNFR1^−/−^), and PI3Kγ-deficient (PI3Kγ^−/−^) mice and respective wild types (WTs) (5-LO^+/+^, CCL3^+/+^, TNFR1^+/+^, and PI3Kγ^+/+^), obtained as previously described (17–21). Mice were obtained from the FIOCRUZ breeding unit, as well as raised and maintained under the same housing conditions. All animal care and experimental protocols were conducted following the guidelines of the Brazilian Council for Care and Use of Experimentation Animals (CONCEA). The Oswaldo Cruz Institute Animal Welfare Committee (CEUA-IOC license number L-011/2015) approved all protocols used in this study.

### *In Vivo* Leptin Treatments

The *in vivo* treatments were performed as previously described (11). Briefly, following the intraperitoneal (i.p.) administration of leptin (0.25, 0.5, 1, and 2 mg/kg, depending on the experiment) or vehicle (sterile, apirogenic saline), animals were euthanized at different time points (1, 6, or 24 h, as specified in each experiment). Alternatively, animals received three i.p. injections of rapamycin (12.5 μg/kg), or vehicle, 12 h before, 15 min before, and 12 h after the injection of leptin or saline, and the peritoneal lavage was harvested after 24 h. This treatment was established by us and was proved to be effective for the inhibition of leptin-induced lipid droplets in peritoneal macrophages (11). We also evaluated the effect of i.p. pretreatments, 15 min prior to leptin treatment, with the phospholipase A_2_ inhibitor, Zileuton (60 μg/cavity), or the LTB_4_ receptor BLT1-specific inhibitor, U-75302 (5 mg/kg). These drugs were administered according to data from previous works from our group and others (22–28). To block CXCL1, the antibody against CXCL1 (3 μg/animal) or the isotype control (diluted in sterile saline) was injected into the peritoneal cavity, 10 min before the leptin injection. After the time specified in each experiment, the peritoneal cells were harvested as follows. The peritoneal cavity was rinsed with HBSS (Hank’s balanced salted solution, 3 mL/cavity), and a volume of approximately 2.5 mL was recovered. Samples were diluted in Turk fluid (2% acetic acid) for total leukocyte counts using Neubauer chambers. Differential leukocyte counting was performed in cytospin smears stained by May–Grünwald–Giemsa, a classical staining for the differential identification of leukocytes (mononuclear cells, neutrophils, and eosinophils) (29). As a control of vascular integrity, we assured that leptin injection does not modify peritoneal lavage protein concentrations There is no difference in the total protein concentration between the samples at 6 h after injection of saline (0.754 ± 0.032 mg/mL) or leptin (0.729 ± 0.048 mg/mL), or at 24 h after injection of saline (0.776 ± 0.014 mg/mL) or leptin (0.749 ± 0.037 mg/mL).

### *In Vitro* Leptin Incubation

The peritoneal cells including the macrophages were obtained from naïve C57Bl/6 mice, by peritoneal lavage with HBSS (5 mL). Cells were transferred to polypropylene tubes (1 × 10^6^ cells/mL) and then incubated with leptin (20 nM) for 4 h, under 37°C, 5% CO_2_ atmosphere in RPMI 1604 medium. After incubation, tubes were centrifuged for supernatant collection. Cell viability was always >85% as determined by Trypan blue exclusion. For the *in vitro* incubation of neutrophils, murine bone marrow neutrophils were obtained as previously described (30). Briefly, the bone marrow from femurs and tibias was washed, and neutrophils were purified in a discontinuous Percoll gradient. Neutrophil viability was greater than 95% as assessed by the Trypan blue exclusion test, and purity was greater than 98% as analyzed by microscopy using Hemacolor staining (Merck, Darmstadt, Germany).

### KC, TNF, Leptin, and LTB_4_ Quantification

Peritoneal lavage or *in vitro* supernatants were analyzed for TNFα, CXCL1/KC, and LTB_4_. TNFα and CXCL1 ELISA were performed with Duo Set kit, according to manufacturer’s protocol (R&D Systems, Minneapolis, MN, USA) and LTB_4_ with enzyme immuno assay (EIA) kit (Cayman Chemical, Ann Arbor, MI, USA). For the investigation of the presence of intracellular LTB_4_, we used the previously described Eicosacell protocol (31). Briefly, leukocytes were recovered from peritoneal cavities and immediately submitted to fixation and permeabilization with 0.5% 1-ethyl-3-(3-dimethylamino-propyl) carbodiimide (Sigma) in HBSS. After that, a common immunodetection protocol was performed using the following antibodies: the primary antibody anti-LTB4 (Cayman Chemical) or irrelevant IgG and the secondary antibody Alexa 488-labeled anti-rabbit IgG. Images were obtained using an Olympus BX51 fluorescence microscope and equipped with a Plan Apo ×100 objective and a DP72 camera (Olympus Optical, Japan) in conjunction with Cell^F^ Imaging Software (Olympus Life Science Europe, Germany).

### Statistical Analysis

Data were reported as the mean ± standard error of the mean (SEM). Data were statistically analyzed by the analysis of variance with Newman–Keuls–Student test, or Student’s *t*-test. Differences were considered to be significant when *p* ≤ 0.05 (see figure legends).

## Results

### Leptin-Induced Selective Neutrophil Recruitment *In Vivo*

**Figure 1:**
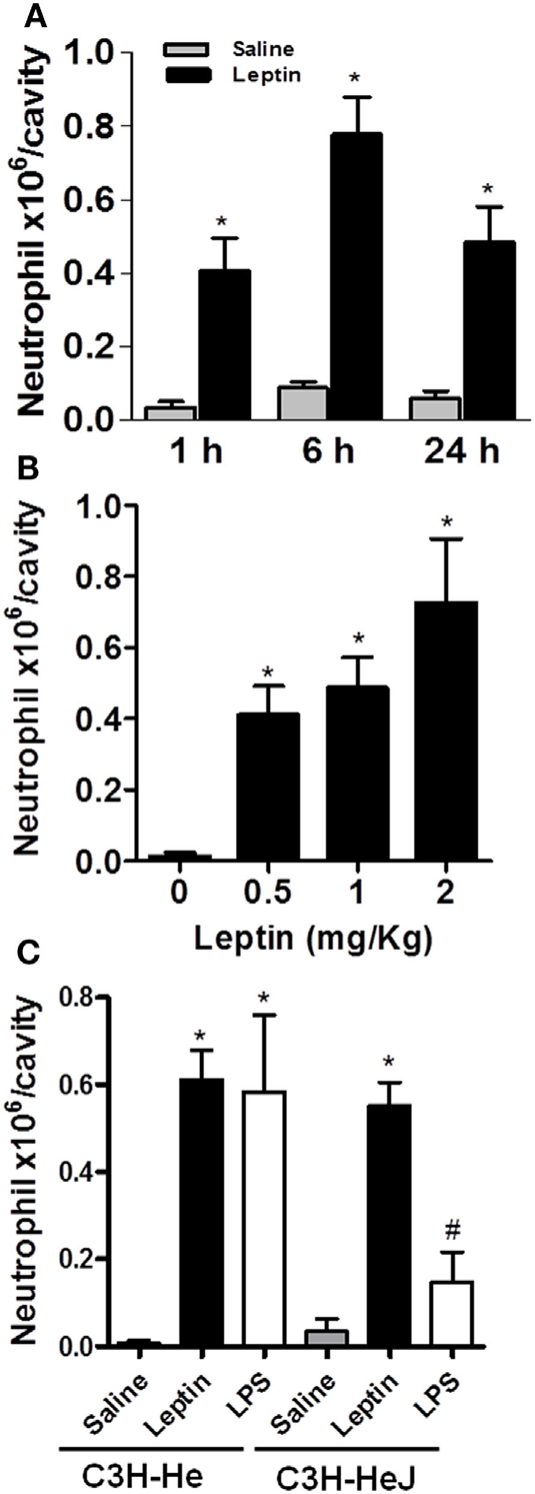
Leptin-induced neutrophil migration. Leptin was injected into the peritoneal cavity of C57Bl/6 mice, and the cells were collected and stained by May–Grünwald–Giemsa for neutrophil recruitment analysis. **(A)** Neutrophils were counted in the peritoneal cavity at 1, 6, and 24 h after leptin (1 mg/kg) injection. **(B)** Different concentrations of leptin (0.5, 1, and 2 mg/kg) led to neutrophils presence in the peritoneal cavity in 24 h. **(C)** Neutrophils accumulated in the peritoneal cavity of C3H-HeJ mice 6 h after leptin injection (1 mg/kg), but not after LPS (80 ng/cavity) or saline injection. Each bar represents the mean ± SEM, *n* = 5–7 **(A,B)** and *n* = 3–6 **(C)**. All experiments were performed at least three times. All data were analyzed by Newman–Keuls–Student test. *Statistically significant (*p* < 0.05) difference between control and stimulated groups. ^#^Statistically significant (*p* < 0.05) difference between C3H-Hej and C3H-He LPS-stimulated groups.

Leptin deficiency is associated with impaired cell-mediated immunity and increased susceptibility to infection (1), and neutrophil chemotaxis is a key component of inflammation and host response to infection. There are very few studies though addressing leptin-inflammatory effects *in vivo*, and the effects of leptin are confounded with the effects of other immunometabolism-modulating factors that are altered in diseases such as infection or obesity (32). To investigate the specific effect of leptin on neutrophil recruitment, here, neutrophil influx was evaluated at different time points after i.p. leptin (1 mg/kg) administration. Leptin-induced neutrophil accumulation in the peritoneal cavity of C57BL/6 mice was significant within 1 h, maximal within 6 h, and sustained for at least until 24 h (Figure 1A). This effect was observed at different leptin concentrations (0.5, 1, and 2 mg/kg) for 24 h (Figure 1B). It is known that after bacterial or LPS stimulation, activated neutrophils present enhanced lipid body numbers, which is considered characteristic of their activation (33–35). In our previous work, the enhancement of lipid droplets in leptin-stimulated macrophages was described (11). However, we did not observe the enhancement of lipid droplets above the basal levels in peritoneal neutrophils after leptin treatment (Figure S1 in Supplementary Material). The i.p. injection of leptin in C3H/HeJ LPS-resistant mice led to significant neutrophil accumulation in the peritoneal cavity within 24 h (Figure 1C). Leptin samples were confirmed negative for LPS contamination by LAL testing (<0.01 UI); therefore, our data indicate that LPS is not involved in the observed leptin response. Moreover, we confirmed the absence of a direct effect of leptin on neutrophils by *in vitro* neutrophil adhesion experiments (Figure S2 in Supplementary Material). Even with very high leptin concentrations (up to 200 nM), no adhesion was observed. In our preparation, we ensured >98% purity of neutrophils as previously described (30).

### Leptin-Induced Neutrophil Migration Is Requisitely Dependent on TNFα

**Figure 2:**
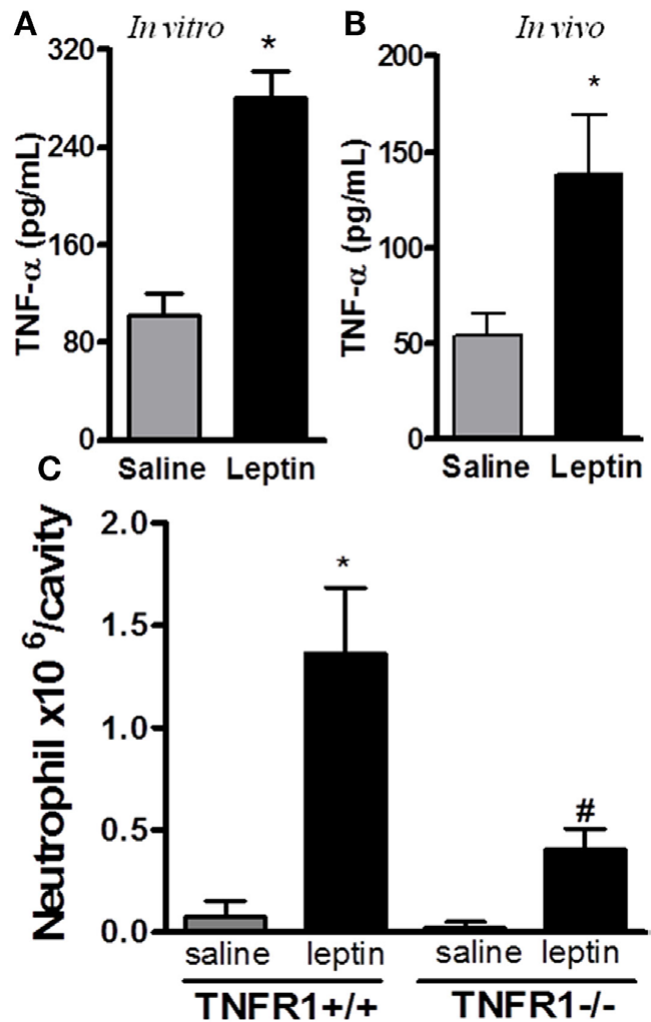
Tumor necrosis factor-alpha (TNFα) participates in leptin-induced neutrophil migration. **(A)** Peritoneal macrophages were stimulated with 20 nM leptin for 4 h *in vitro*, before TNFα measurement in the culture supernatant. **(B)** C57Bl/6 mice were injected intraperitoneally with leptin (1 mg/kg) *in vivo* and, 6 h afterward, TNFα was measured in the peritoneal washing supernatant. **(C)** Tumor necrosis factor receptor 1 (TNFR1^−/−^) or TNFR1^+/+^ mice received leptin (1 mg/kg) or saline i.p. injection. The analysis of neutrophil recruitment was performed at 24 h after leptin administration. Each bar represents the mean ± SEM, *n* = 4–5. Experiments A and B were performed at least three times. Data were analyzed by analysis by Student’s *t*-test **(A,B)** and by Newman–Keuls–Student test. *Statistically significant difference (*p* < 0.05) between leptin-stimulated and saline groups. ^#^Statistically significant difference (*p* < 0.05) between TNFR1^−/−^ and TNFR1^+/+^ leptin-stimulated groups.

It had been previously demonstrated that leptin indirectly activates human neutrophils (10). This work showed that neutrophil CD11b expression can be enhanced in response to TNFα produced by leptin-stimulated monocytes *in vitro*. We confirmed that leptin induced TNFα production by the peritoneal cells *in vitro* (Figure 2A). Moreover, leptin injection into the peritoneal cavity induced *in vivo* TNFα production (Figure 2B). In order to investigate the role of TNFα receptor on leptin-induced neutrophil recruitment *in vivo*, TNFR1 knockout mice (TNFR1^−/−^) or WT (TNFR1^+/+^) animals (C57Bl/6) were used. As demonstrated in Figure 2C, leptin was unable to induce neutrophil accumulation in TNFR1^−/−^ mice, but interestingly, TNFR1 is not necessary for the effect of leptin in inducing lipid droplets in macrophages (Figure S3 in Supplementary Material). Leptin induced lipid body formation in peritoneal macrophages from TNFR1^−/−^ mice, similar to what was observed in WT (TNFR1^+/+^ mice). Thus, leptin-induced neutrophil migration is dependent on TNFR1 signaling, but macrophage lipid body formation occurs independently of this TNFα receptor.

### Leptin-Induced Neutrophil Recruitment Is Dependent on the PI3Kγ Pathway

**Figure 3:**
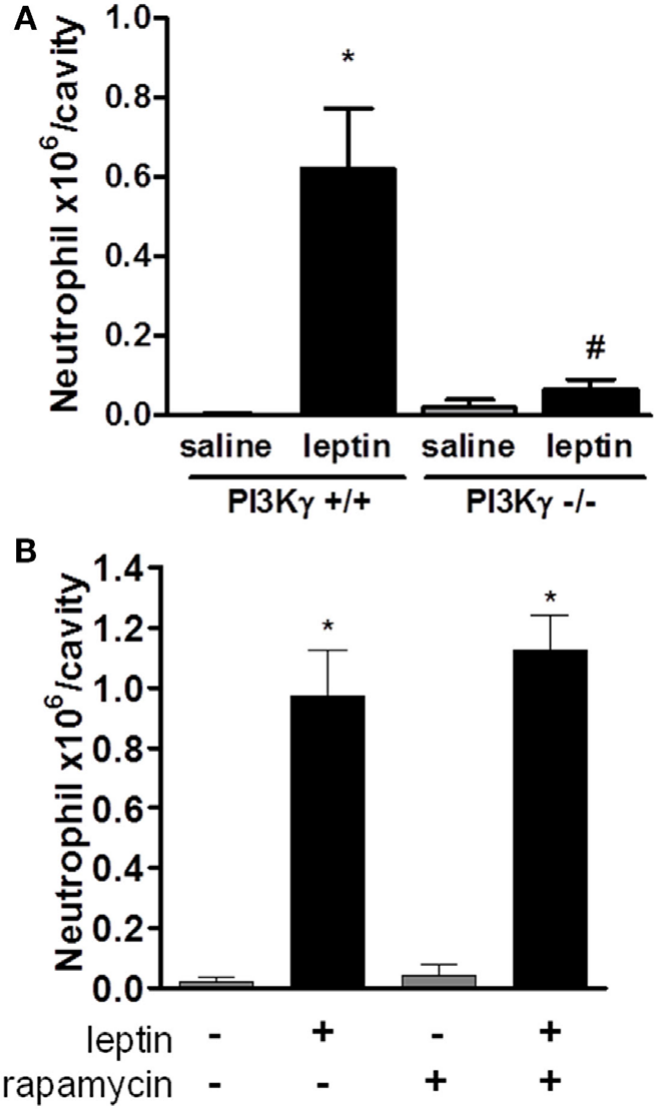
Leptin-induced neutrophil migration is dependent on PI3Kγ but independent of mammalian target of rapamycin (mTOR) signaling. **(A)** PI3Kγ^+/+^ or PI3Kγ^−/−^ mice were injected intraperitoneally with leptin (1 mg/kg) or saline. Neutrophils were counted in the peritoneal wash at 24 h after leptin injection. **(B)** C57Bl/6 mice received rapamycin treatment (12.5 µg/kg per injection) 12 h before, 15 min before, and 12 h after leptin injection. Each bar represents the mean ± SEM, *n* = 3–6 **(A)** and 6–8 **(B)**. The experiments were performed at least three times. Data were analyzed by Newman–Keuls–Student test. *Statistically significant difference (*p* < 0.05) between leptin-stimulated and saline groups. ^#^Statistically significant difference (*p* < 0.05) between PI3Kγ^−/−^ and PI3Kγ^+/+^ leptin-stimulated groups.

Several studies have demonstrated the importance of PI3K, specifically PI3Kγ, in leukocyte migration and activation *in vivo* (19, 36–39). To determine the role of PI3Kγ in the acute response induced by leptin, we examined neutrophil in WT mice or mice genetically deficient in PI3Kγ (PI3Kγ^−/−^), 24 h after leptin i.p. administration. Leptin-induced accumulation of neutrophils in the peritoneal cavity was completely inhibited in PI3Kγ^−/−^ mice in comparison to that in WT control animals (Figure 3A). Thus, the PI3Kγ-signaling pathway is required for leptin-induced neutrophil recruitment.

### Leptin-Induced Neutrophil Recruitment Is Independent on mTOR Pathway

We showed previously that mTOR was required for the effects of leptin on lipid droplets formation in macrophages both *in vitro* and *in vivo* (11). Others reported that the mTOR pathway was important for GM-CSF-dependent neutrophil activation *in vitro* (40). We investigated here the functional role of the mTOR pathway for the *in vivo* leptin-induced neutrophil activation and recruitment. We used the previously described treatment with rapamycin, a specific inhibitor and probe for mTOR activity (11, 41). The treatment of mice with rapamycin failed to modify leptin-induced neutrophil accumulation after 24 h (Figure 3B).

### Leptin-Induced Neutrophil Migration Is Independent of LTB_4_

**Figure 4:**
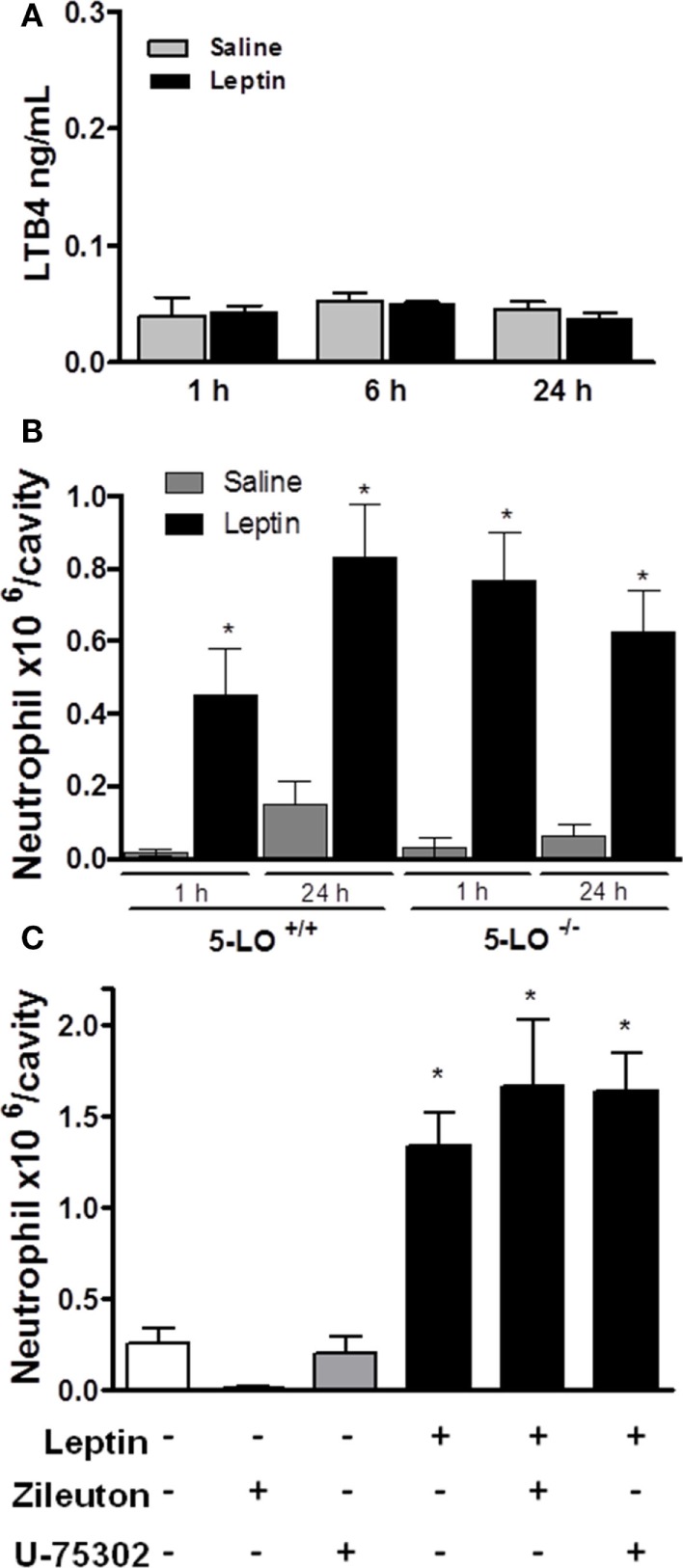
Leptin-induced neutrophil migration is independent of LTB_4_ signaling. **(A)** C57Bl/6 mice were injected intraperitoneally with leptin (1 mg/kg), and after 24 h LTB_4_ EIA was performed in the peritoneal washing supernatant. **(B)** 5-LO^+/+^ or 5-LO^−/−^ mice were injected intraperitoneally with leptin (1 mg/kg) or saline. Neutrophils were counted in the peritoneal wash at 24 h after leptin injection. **(C)** C57Bl/6 mice were pre-treated with U-75302 (5 mg/kg) or Zileuton (60 µg/animal), 10 min before receiving leptin injection (1.5 mg/kg). Neutrophils were counted in peritoneal wash, 3 h after leptin injection. Each bar represents the mean ± SEM, *n* = 5–8. The experiments were performed at least three times. Data were analyzed by Newman–Keuls–Student test. *Statistically significant difference (*p* < 0.05) between leptin and saline. There are no statistically significant differences (*p* > 0.05) between 5-LO^−/−^ and 5-LO^+/+^ leptin-stimulated groups.

It had been shown that the lipid mediator LTB_4_ directly exerts a chemoattractive effect on neutrophils in inflammatory conditions (42, 43). We performed several *in vivo* experiments in order to evaluate the role of LTB_4_ in neutrophil migration stimulated by leptin. First, an EIA assay was performed to detect LTB_4_ presence on peritoneal supernatants obtained after leptin i.p. injection. There was no difference in the concentration of LTB_4_ between saline and 1-, 6-, or 24-h leptin-stimulated groups (Figure 4A). It could be that LTB_4_ was produced but not released from the cell; therefore, we performed the Eicosacell assay. We did not observe intracellularly retained LTB_4_ in the peritoneal cells submitted to the Eicosacell assay as performed before by our group (data not shown) (31). We also investigated the importance of the LTB_4_ synthesis enzyme, 5-LO for neutrophil migration. Therefore, 5-LO^+/+^ or 5-LO^−/−^ mice were injected with leptin in the peritoneal cavity (1 mg/kg), and neutrophil migration was investigated. As shown in Figure 4B, leptin induced neutrophil migration (24 h) in 5-LO knockout mice. The pharmacological inhibition of LTB_4_ synthesis by Zileuton (60 µg/animal i.p.) and signaling on the receptor BTL1 by U-75302 (5 mg/kg i.p.) were tested. Figure 4C shows that peritoneal neutrophils migrate in response to leptin injection, despite pharmacological pretreatments. These results demonstrate that LTB_4_ does not participate in leptin-induced neutrophil recruitment.

### Leptin-Induced Neutrophil Migration Is Dependent on CXCL1

**Figure 5:**
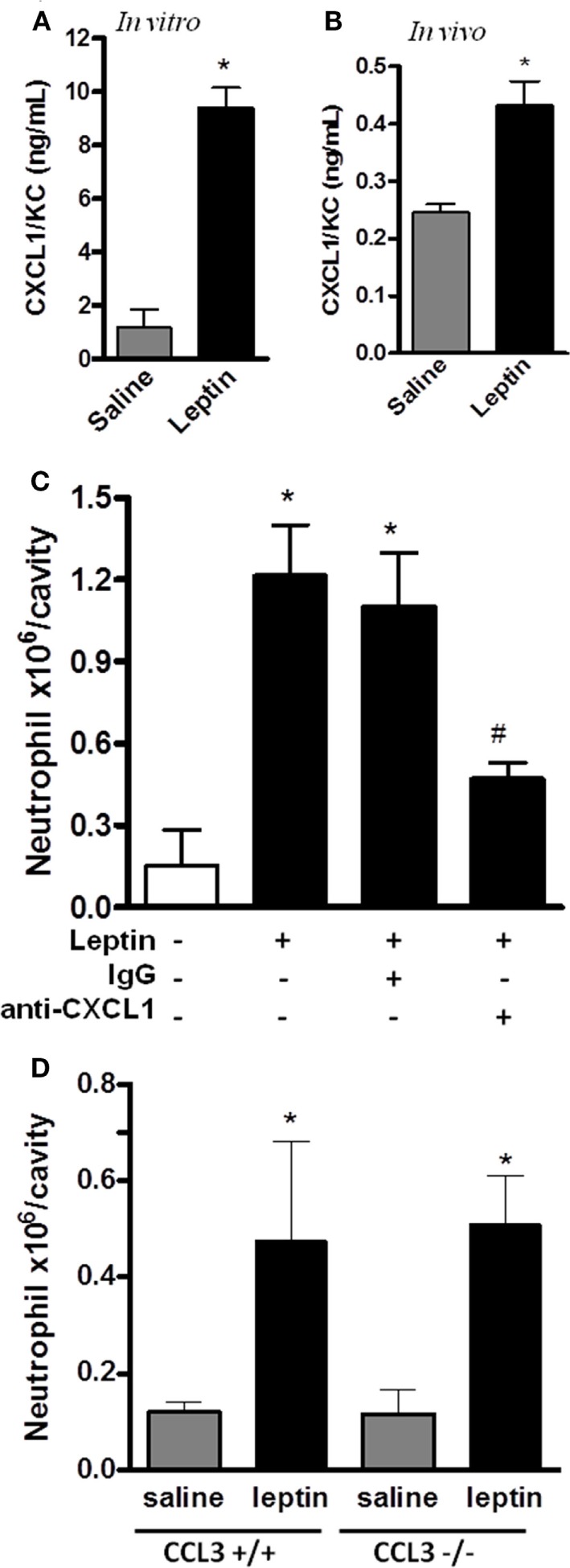
Leptin-induced neutrophil migration is dependent on CXCL1/KC signaling but independent of CCL3 signaling. **(A)** Peritoneal macrophages were stimulated with 20 nM leptin *in vitro*, for 4 h, and CXCL1 was measured in the culture supernatant. **(B)** C57Bl/6 mice were injected intraperitoneally with leptin (1 mg/kg), *in vivo*, and after 6 h, CXCL1 was measured in the peritoneal washing supernatants. **(C)** C57BL/6 mice were treated with either non-specific IgG or anti-CXCL1 antibodies (3 microgram/cavity i.p.), 10 min before leptin injection (1 mg/kg; i.p.). Neutrophils were counted in the peritoneal wash at 4 h after leptin injection. **(D)** CCL3^+/+^ or CCL3^−/−^ mice were injected intraperitoneally with leptin (1 mg/kg) or saline. Neutrophils were counted in the peritoneal wash at 24 h after leptin injection. Each bar represents the mean ± SEM, *n* = 4–6 **(A,B)**, *n* = 7–9 **(C)**, and *n* = 5–8 **(D)**. The experiments were performed at least three times. Data were analyzed by Student’s *t*-test **(A,B)** or by Newman–Keuls–Student test **(C,D)**. *Statistically significant differences (*p* < 0.05) between leptin-stimulated and saline groups. ^#^Statistically significant differences (*p* < 0.05) between anti-CXCL1 plus leptin group and leptin-stimulated group. There is no statistically significant difference (*p* > 0.05) between CCL3^−/−^ and CCL3^+/+^ leptin-stimulated groups.

CXCL1 is known to mediate the neutrophil migration through the activation of CXCR2, a PI3Kγ-dependent receptor (44). Neutrophil migration can also be promoted by CCL3/MIP-1α signaling through CCR1, a PI3Kγ-dependent receptor (45). Because our result in Figure 3A showed that leptin-induced neutrophil recruitment is dependent on PI3Kγ, we investigated the chemokines CXCL1 and CCL3 as possible mediators for this effect. We observed that CXCL1 is secreted by the peritoneal cells stimulated by leptin both *in vitro* and *in vivo* (Figures 5A,B). Anti-CXCL1-neutralizing antibodies were used *in vivo* as a pretreatment before leptin injection. A partial but significant inhibition of neutrophil migration to peritoneal cavity was observed (Figure 5C). We investigated the participation of the chemokine CCL3 using the CCL3 knockout mice (CCL3^−/−^). Mice were injected with leptin i.p. and, as seen in Figure 5D, neutrophil recruitment was not altered when compared to WT mice (CCL3^+/+^). Taken together, our data show that leptin activates the peritoneal cells to induce neutrophil migration in a specific CXCL1-dependent manner.

## Discussion

We demonstrated here that the *in vivo* administration of leptin significantly induces neutrophil recruitment to the peritoneal cavity. We propose here that this is an indirect effect of leptin, which requires peritoneal resident cell activation with the production of TNFα and CXCL1, mostly by resident monocytes/macrophages. It was shown before that leptin induces TNFα production by macrophages, and under different inflammatory stimuli, peritoneal macrophages produce TNFα and CXCL1 that are important for neutrophil migration (10, 16, 46). Leptin contribution to the modulation of neutrophil activation has been controversial because neutrophils present LepRa but lack LepRb (10, 47). It was suggested that leptin could stimulate chemotaxis and the release of hydrogen peroxide by human neutrophils (48). Others demonstrated that leptin could only indirectly trigger increased CD11b expression on human neutrophils by the induction of TNFα release from monocytes *in vitro*, and highly purified neutrophils (more than 98%) do not produce TNF under leptin stimulation (10). It was also suggested that leptin could inhibit neutrophil apoptosis in a LepRa-dependent manner (49). More recently, it was demonstrated that only extremely high concentrations of leptin could drive direct changes on neutrophil activation or the inhibition of apoptosis (above 3–25 µg/mL; 200–1,600 nM) (50). Kamp et al. concluded that there is no convincing evidence that physiological leptin concentrations could directly activate neutrophils. Accordingly, we found no significant activation of adhesion, even with 200 nM (Figure S2 in Supplementary Material). These previous studies to evaluate the neutrophil capacity to migrate were performed *in vitro*. We decided to study the *in vivo* mechanisms for neutrophil migration under exogenous leptin administration. Our data show *in vivo* leptin-induced neutrophil recruitment and suggest an indirect activation of other resident cells. Lipid droplet formation is associated with leukocyte activation in different inflammatory models (33, 35, 51, 52). Interestingly, we found that there was no enhanced lipid droplet formation in leptin-dependent-migrating neutrophils (Figure S1 in Supplementary Material). We suggest here that leptin effects are not sufficient for the complete activation of neutrophils *in vivo*.

The contribution of LTB_4_ to inflammation progression is well known. This lipid mediator is both an important neutrophil chemotaxis inducer, the activator of phagocytosis, and the main eicosanoid produced by neutrophils under diverse pro-inflammatory stimuli ((24, 53–57)). We had previously shown that leptin-stimulated peritoneal macrophages have a higher capacity to produce LTB_4_ following calcium ionophore activation (11). The absence of LTB_4_ in the peritoneal cavity, shown in Figure 4A, provides evidence that the migrating neutrophils, after leptin stimulation, are not fully activated. Moreover, leptin induced neutrophil recruitment despite the genetic 5-LO null background, the pharmacological inhibition of 5-LO, or the pharmacological blockage of BLT1 (Figure 4). These results rule out an involvement of LTB_4_ in leptin-induced neutrophil activation and recruitment *in vivo*.

It was known that TNFα induces CD11b expression on neutrophils, an important integrin component that participates in intraluminal adhesion and crawling in the site of inflammation (10, 58). We show here that the leptin effect on neutrophil migration is mediated by TNFα signaling through TNFR1. Indeed, leptin failed to recruit neutrophils to the peritoneal cavity in mice genetically deficient in TNFR1. An indirect action of leptin on neutrophils mediated by TNFα supports the involvement of this pathway in acute inflammatory responses. Chemokines, produced by resident cells under TNFα stimulation, such as the CXCL1/KC, the mouse analog to human IL-8, have been shown to induce the recruitment of neutrophils to inflammatory sites (16). Moreover, leptin induces CXCL1 in epithelial cells (59). Here, we show that resident peritoneal cells secrete CXCL1 after leptin stimulation, both *in vivo* and *in vitro*, and CXCL1 neutralization partially inhibits neutrophil migration. We conclude that leptin-induced neutrophil recruitment is dependent on the CXCL1 chemokine produced by the peritoneal cells. It was recently described that adipose stromal cells can be recruited from white adipose tissue to tumor environments through CXCL1 signaling, suggesting that the increased aggressiveness of certain cancers is linked to white adipose tissue overgrowth in obesity (60). Our data offer a possible explanation for the role of leptin in tumor cell recruitment during obesity, when high levels of leptin production by white adipose tissue are observed. The PI3Kγ-signaling pathway was shown to be relevant for neutrophil recruitment induced by CXCL1 *in vivo* in specific sites of inflammatory stimuli. Neutrophil recruitment to bronchoalveolar space induced by CXCL1 was prevented in PI3Kγ^−/−^ mice (44). However, these authors showed that the administration of CXCL1 in the cremaster muscle of PI3Kγ^−/−^ or WT mice induced similar increases in neutrophil rolling, adhesion, and transmigration. Our data show that leptin-induced neutrophil migration to the peritoneal cavity is prevented in PI3Kγ^−/−^ mice. We suggest that this may be caused by CXCL1 signaling through PI3Kγ, since PI3K-regulatory subunits differentially participate in neutrophil migration either *in vivo* and *in vitro* (61). It may also reflect the combination of PI3Kγ activation in endothelium and neutrophils, which is required for leukocyte trafficking to sites of inflammation (62). Based on our findings and previous published data, we conclude that PI3Kγ have both direct and indirect effects on neutrophil recruitment, since PI3Kγ is part of the signaling pathway activated by CXCL1 in neutrophils and is important for the leptin-induced activation of macrophages (11, 44, 63).

Other chemokines were shown to be important for neutrophil recruitment, such as CCL3, and both insulin and TNFα could prime the neutrophils for CCL3/MIP-1α-dependent migration (64, 65). Different from insulin, we observed that the leptin effect was not dependent on CCL3 since neutrophil migration was maintained on the CCL3-deficient mice (Figure 5C).

We previously demonstrated that leptin induced lipid droplet formation in peritoneal macrophages in a PI3K/mTOR-dependent manner (11). Interestingly, the lipid droplet induction by leptin on resident peritoneal macrophages is not dependent on TNFα signaling because this effect was maintained in the TNFR1^−/−^ (Figure S3 in Supplementary Material). Therefore, leptin induces TNFα- and CXCL1-dependent neutrophil migration, by a distinct pathway from lipid droplet formation in the peritoneal cells.

In the immune system, mTOR is involved in signaling downstream from different inflammatory stimuli. Phosphatidic acid and LPS are examples of leukocyte activation inducers that depend on mTOR activation (66–68), and also GM-CSF-induced neutrophil migration was shown to be inhibited by rapamycin (40). The mTOR pathway is important for the PAF-induced activation of neutrophils, inducing the translation of the cytokine IL-6 receptor (69). Nevertheless, we showed here that the indirect effect of leptin-induced migration of neutrophils is independent of the TORC1 pathway (rapamycin-inhibited mTOR complex). Rapamycin is a proapoptotic drug for different cells including neutrophils. Since rapamycin does not alter neutrophil accumulation after 24 h (Figure 3), we suggest that the mTOR-dependent inhibition of apoptosis is not the main effect of leptin on neutrophils *in vivo*.

It has been shown that LPS-induced systemic inflammation presents neutrophils transmigration into the brain in a leptin-dependent manner (70, 71). Our data are in agreement to these *in vivo* results, but we show that leptin can promote *in vivo* neutrophil migration independently of LPS-inflammatory response. In a lung injury model, the intranasal administration of leptin counteracted some LPS effects in neutrophils (72). Such results are in apparent contrast with our hypothesis and data. We think that these results are due to tissue-specific effects. We evaluated the peritoneal cavity, while Landgraf et al. analyzed lung tissue, where leptin can directly activate the alveolar epithelium. We recently studied the direct effects of leptin intestinal epithelial cells and observed an enhancement of TGFβ that could be responsible for inhibitory effects on the tissue macrophages (59). It has also been suggested that there may be different subsets of neutrophils (73). These different neutrophil subsets may then have diverse responses toward direct or indirect leptin stimulation. Therefore, it would be very interesting to further explore the differential effects of leptin on leukocytes migration in distinct tissues, administration sites, and different neutrophil subsets.

Our data reveal the importance of leptin for neutrophil migration to sites of inflammation. We argue that it is crucial to understand these effects in great detail, in particular because of the prescription of recombinant leptin in different human conditions.

## Ethics Statement

All animal care and experimental protocols were conducted following the guidelines of the Brazilian Council for Care and Use of Experimentation Animals (CONCEA). The Oswaldo Cruz Institute Animal Welfare Committee (CEUA_IOC, L-011/2015) approved all protocols used in this study.

## Author Contributions

All authors had critically revised and approved the final version of the manuscript. GS-A and CM-M performed the conception, designed and performed the experiments, analyzed and interpreted data, and wrote the manuscript draft. HB, PA, TG, SL, IH, and BW participated in the data acquisition, analysis, and interpretation of the data. HC-F-N, PB, and CB-M participated in the conception, design, analysis, and interpretation of the work.

## Conflict of Interest Statement

The authors declare that the research was conducted in the absence of any commercial or financial relationships that could be construed as a potential conflict of interest.

## Abbreviations

5-LO, 5-lipoxygenase; ADRP, adipose differentiation-related protein; EIA, enzyme immuno assay; mTOR, mammalian target of rapamycin; LTB_4_, leukotriene B_4_; PLA_2_, phospholipase A_2_; TNFR1, tumor necrosis factor receptor 1.

## References

[1] Matarese G Moschos S Mantzoros CS. Leptin in immunology. J Immunol (2005) 174:3137–42. 10.4049/jimmunol.174.6.3137 15749839

[2] Procaccini C De Rosa V Galgani M Carbone F Cassano S Greco D Leptin-induced mTOR activation defines a specific molecular and transcriptional signature controlling CD4+ effector T cell responses. J Immunol (2012) 189:2941–53. 10.4049/jimmunol.1200935 22904304

[3] Moon H-S Matarese G Brennan AM Chamberland JP Liu X Fiorenza CG Efficacy of Metreleptin in obese patients with type 2 diabetes: cellular and molecular pathways underlying leptin tolerance. Diabetes (2011) 60:1647–56. 10.2337/db10-1791 21617185 PMC3114380

[4] Polyzos SA Mantzoros CS Leptin in health and disease: facts and expectations at its twentieth anniversary. Metabolism (2015) 64:5–12. 10.1016/j.metabol.2014.10.017 25467841

[5] Shetty GK Matarese G Magkos F Moon H-S Liu X Brennan AM Leptin administration to overweight and obese subjects for 6 months increases free leptin concentrations but does not alter circulating hormones of the thyroid and IGF axes during weight loss induced by a mild hypocaloric diet. Eur J Endocrinol (2011) 165:249–54. 10.1530/EJE-11-0252 21602313 PMC3159386

[6] Wabitsch M Funcke J-B von Schnurbein J Denzer F Lahr G Mazen I Severe early-onset obesity due to bioinactive leptin caused by a p.N103K mutation in the leptin gene. J Clin Endocrinol Metab (2015) 100:3227–30. 10.1210/jc.2015-2263 26186301 PMC4570156

[7] Chou K Perry CM. Metreleptin: first global approval. Drugs (2013) 73:989–97. 10.1007/s40265-013-0074-7 23740412

[8] La Cava A Matarese G The weight of leptin in immunity. Nat Rev Immunol (2004) 4:371–9. 10.1038/nri1350 15122202

[9] Abella V Scotece M Conde J Pino J Gonzalez-Gay MA Gómez-Reino JJ Leptin in the interplay of inflammation, metabolism and immune system disorders. Nat Rev Rheumatol (2017) 13:100–9. 10.1038/nrrheum.2016.209 28053336

[10] Zarkesh-Esfahani H Pockley AG Wu Z Hellewell PG Weetman AP Ross RJM Leptin indirectly activates human neutrophils *via* induction of TNF-. J Immunol (2004) 172:1809–14. 10.4049/jimmunol.172.3.1809 14734764

[11] Maya-Monteiro CM Almeida PE D’Avila H Martins AS Rezende AP Castro-Faria-Neto H Leptin induces macrophage lipid body formation by a phosphatidylinositol 3-kinase- and mammalian target of rapamycin-dependent mechanism. J Biol Chem (2008) 283:2203–10. 10.1074/jbc.M706706200 18039669

[12] Mancuso P Huffnagle GB Olszewski M Phipps J Peters-Golden M. Leptin corrects host defense defects after acute starvation in murine pneumococcal pneumonia. Am J Respir Crit Care Med (2006) 173:212–8. 10.1164/rccm.200506-909OC 16210671

[13] Behnes M Brueckmann M Lang S Putensen C Saur J Borggrefe M Alterations of leptin in the course of inflammation and severe sepsis. BMC Infect Dis (2012) 12:217. 10.1186/1471-2334-12-217 22973876 PMC3462137

[14] Nathan C. Neutrophils and immunity: challenges and opportunities. Nat Rev Immunol (2006) 6:173–82. 10.1038/nri1785 16498448

[15] Kolaczkowska E Kubes P. Neutrophil recruitment and function in health and inflammation. Nat Rev Immunol (2013) 13:159–75. 10.1038/nri3399 23435331

[16] Vieira SM Lemos HP Grespan R Napimoga MH Dal-Secco D Freitas A A crucial role for TNF-alpha in mediating neutrophil influx induced by endogenously generated or exogenous chemokines, KC/CXCL1 and LIX/CXCL5. Br J Pharmacol (2009) 158:779–89. 10.1111/j.1476-5381.2009.00367.x 19702783 PMC2765597

[17] Cook D Beck M Coffman T Kirby S Sheridan J Pragnell I Requirement of MIP-1 alpha for an inflammatory response to viral infection. Science (1995) 269(5230):1583–5. 10.1126/science.7667639 7667639

[18] Goulet JL Snouwaert JN Latour AM Coffman TM Koller BH. Altered inflammatory responses in leukotriene-deficient mice. Proc Natl Acad Sci U S A (1994) 91(26):12852–6. 10.1073/pnas.91.26.12852 7809134 PMC45538

[19] Sasaki T Function of PI3K in thymocyte development, T cell activation, and neutrophil migration. Science (2000) 287:1040–6. 10.1126/science.287.5455.1040 10669416

[20] Watson J Riblet R. Genetic control of responses to bacterial lipopolysaccharides in mice. I. Evidence for a single gene that influences mitogenic and immunogenic responses to lipopolysaccharides. J Exp Med (1974) 140:1147–61. 10.1084/jem.140.5.1147 4138849 PMC2139714

[21] Zheng L Fisher G Miller RE Peschon J Lynch DH Lenardo MJ. Induction of apoptosis in mature T cells by tumour necrosis factor. Nature (1995) 377:348–51. 10.1038/377348a0 7566090

[22] Gomides LF Duarte ID Ferreira RG Perez AC Francischi JN Klein A. Proteinase-activated receptor-4 plays a major role in the recruitment of neutrophils induced by trypsin or carrageenan during pleurisy in mice. Pharmacology (2012) 89:275–82. 10.1159/000337378 22517275

[23] Li X Fu H Yi W Zhao Y Wang J Li J Dual role of leukotriene B4 receptor type 1 in experimental sepsis. J Surg Res (2015) 193:902–8. 10.1016/j.jss.2014.09.013 25439504

[24] Monteiro APT Pinheiro CS Luna-Gomes T Alves LR Maya-Monteiro CM Porto BN Leukotriene B4 mediates neutrophil migration induced by heme. J Immunol (2011) 186:6562–7. 10.4049/jimmunol.1002400 21536805

[25] Rao TS Currie JL Shaffer AF Isakson PC *In vivo* characterization of zymosan-induced mouse peritoneal inflammation. J Pharmacol Exp Ther (1994) 269:917–25. 8014878

[26] Rao TS Yu SS Djuric SW Isakson PC Phorbol ester-induced dermal inflammation in mice: evaluation of inhibitors of 5-lipoxygenase and antagonists of leukotriene B4 receptor. J Lipid Mediat Cell Signal (1994) 10:213–28. 7812673

[27] Silva AR de Assis EF Caiado LFC Marathe GK Bozza MT McIntyre TM Monocyte chemoattractant protein-1 and 5-lipoxygenase products recruit leukocytes in response to platelet-activating factor-like lipids in oxidized low-density lipoprotein. J Immunol (2002) 168:4112–20. 10.4049/jimmunol.168.8.4112 11937571

[28] Sun M Wang R Han Q. Inhibition of leukotriene B4 receptor 1 attenuates lipopolysaccharide-induced cardiac dysfunction: role of AMPK-regulated mitochondrial function. Sci Rep (2017) 7:44352. 10.1038/srep44352 28290498 PMC5349523

[29] D’Avila H Roque NR Cardoso RM Castro-Faria-Neto HC Melo RCN Bozza PT. Neutrophils recruited to the site of *Mycobacterium bovis* BCG infection undergo apoptosis and modulate lipid body biogenesis and prostaglandin E production by macrophages. Cell Microbiol (2008) 10:2589–604. 10.1111/j.1462-5822.2008.01233.x 18771558

[30] Schymeinsky J Sindrilaru A Frommhold D Sperandio M Gerstl R Then C The Vav binding site of the non-receptor tyrosine kinase Syk at Tyr 348 is critical for beta2 integrin (CD11/CD18)-mediated neutrophil migration. Blood (2006) 108:3919–27. 10.1182/blood-2005-12-030387 16882714

[31] Bandeira-Melo C Weller PF Bozza PT. Eicosacell – an immunofluorescent-based assay to localize newly synthesized eicosanoid lipid mediators at intracellular sites. Methods Mol Biol (2011) 689:163–81. 10.1007/978-1-60761-950-5_10 21153792 PMC3679533

[32] Trottier MD Naaz A Kacynski K Yenumula PR Fraker PJ. Functional capacity of neutrophils from class III obese patients. Obesity (2012) 20:1057–65. 10.1038/oby.2011.354 22158006

[33] Bozza PT Magalhães KG Weller PF. Leukocyte lipid bodies – biogenesis and functions in inflammation. Biochim Biophys Acta (2009) 1791:540–51. 10.1016/j.bbalip.2009.01.005 19416659 PMC2693476

[34] Melo RCN Sabban A Weller PF. Leukocyte lipid bodies: inflammation-related organelles are rapidly detected by wet scanning electron microscopy. J Lipid Res (2006) 47:2589–94. 10.1194/jlr.D600028-JLR200 16940552

[35] Weller PF Ackerman SJ Nicholson-Weller A Dvorak AM. Cytoplasmic lipid bodies of human neutrophilic leukocytes. Am J Pathol (1989) 135:947–59. 2510521 PMC1880101

[36] Hirsch E Katanaev VL Garlanda C Azzolino O Pirola L Silengo L Central role for G protein-coupled phosphoinositide 3-kinase γ in inflammation. Science (2000) 287:1049–53. 10.1126/science.287.5455.1049 10669418

[37] Rickert P Leukocytes navigate by compass: roles of PI3Kγ and its lipid products. Trends Cell Biol (2000) 10:466–73. 10.1016/S0962-8924(00)01841-9 11050418 PMC2819116

[38] Servant G Polarization of chemoattractant receptor signaling during neutrophil chemotaxis. Science (2000) 287:1037–40. 10.1126/science.287.5455.1037 10669415 PMC2822871

[39] Pinho V Souza DG Barsante MM Hamer FP De Freitas MS Rossi AG Phosphoinositide-3 kinases critically regulate the recruitment and survival of eosinophils *in vivo*: importance for the resolution of allergic inflammation. J Leukoc Biol (2005) 77:800–10. 10.1189/jlb.0704386 15860799

[40] Gomez-Cambronero J. Rapamycin inhibits GM-CSF-induced neutrophil migration. FEBS Lett (2003) 550:94–100. 10.1016/S0014-5793(03)00828-7 12935893 PMC3074563

[41] Shamji AF Nghiem P Schreiber SL. Integration of growth factor and nutrient signaling: implications for cancer biology. Mol Cell (2003) 12:271–80. 10.1016/j.molcel.2003.08.016 14536067

[42] Oyoshi MK He R Li Y Mondal S Yoon J Afshar R Leukotriene B4-driven neutrophil recruitment to the skin is essential for allergic skin inflammation. Immunity (2012) 37:747–58. 10.1016/j.immuni.2012.06.018 23063331 PMC3478399

[43] Pazos MA Pirzai W Yonker LM Morisseau C Gronert K Hurley BP. distinct cellular sources of hepoxilin A _3_ and leukotriene B _4_ are used to coordinate bacterial-induced neutrophil transepithelial migration. J Immunol (2015) 194:1304–15. 10.4049/jimmunol.1402489 25548217 PMC4297725

[44] Pinho V de Castro Russo R Amaral FA de Sousa LP Barsante MM de Souza DG Tissue- and stimulus-dependent role of phosphatidylinositol 3-kinase isoforms for neutrophil recruitment induced by chemoattractants *in vivo*. J Immunol (2007) 179:7891–8. 10.4049/jimmunol.179.11.7891 18025236

[45] Reichel CA Puhr-Westerheide D Zuchtriegel G Uhl B Berberich N Zahler S C-C motif chemokine CCL3 and canonical neutrophil attractants promote neutrophil extravasation through common and distinct mechanisms. Blood (2012) 120:880–90. 10.1182/blood-2012-01-402164 22674804

[46] Lee SM Choi HJ Oh CH Oh JW Han JS Leptin increases TNF-α expression and production through phospholipase D1 in Raw 264.7 cells. PLoS One (2014) 9(7):e102373 10.1371/journal.pone.0102373 25047119 PMC4105621

[47] Caldefie-Chezet F Poulin A Tridon A Sion B Vasson M-P. Leptin: a potential regulator of polymorphonuclear neutrophil bactericidal action? J Leukoc Biol (2001) 69:414–8. 10.1189/jlb.69.3.414 11261788

[48] Caldefie-Chezet F Poulin A Vasson M-P. Leptin regulates functional capacities of polymorphonuclear neutrophils. Free Radic Res (2003) 37:809–14. 10.1080/1071576031000097526 14567439

[49] Bruno A Conus S Schmid I Simon H-U. Apoptotic pathways are inhibited by leptin receptor activation in neutrophils. J Immunol (2005) 174:8090–6. 10.4049/jimmunol.174.12.8090 15944317

[50] Kamp VM Langereis JD van Aalst CW van der Linden JA Ulfman LH Koenderman L. Physiological concentrations of leptin do not affect human neutrophils. PLoS One (2013) 8(9):e73170. 10.1371/journal.pone.0073170 24066032 PMC3774682

[51] Bozza PT Melo RCN Bandeira-Melo C. Leukocyte lipid bodies regulation and function: contribution to allergy and host defense. Pharmacol Ther (2007) 113:30–49. 10.1016/j.pharmthera.2006.06.006 16945418

[52] Weller PF Leukocyte lipid bodies – structure and functions as “Eicosasomes.”. Trans Am Clin Climatol Assoc (2016) 127:328–40. 28066068 PMC5216467

[53] Palmblad J Malmsten CL Udén AM Rådmark O Engstedt L Samuelsson B. Leukotriene B4 is a potent and stereospecific stimulator of neutrophil chemotaxis and adherence. Blood (1981) 58:658–61. 6266432

[54] Mancuso P Standiford TJ Marshall T Peters-Golden M. 5-Lipoxygenase reaction products modulate alveolar macrophage phagocytosis of *Klebsiella pneumoniae*. Infect Immun (1998) 66:5140–6. 9784515 10.1128/iai.66.11.5140-5146.1998 PMC108641

[55] Mancuso P Nana-Sinkam P Peters-Golden M. Leukotriene B4 augments neutrophil phagocytosis of *Klebsiella pneumoniae*. Infect Immun (2001) 69:2011–6. 10.1128/IAI.69.4.2011-2016.2001 11254552 PMC98124

[56] Chou RC Kim ND Sadik CD Seung E Lan Y Byrne MH Lipid-cytokine-chemokine cascade drives neutrophil recruitment in a murine model of inflammatory arthritis. Immunity (2010) 33:266–78. 10.1016/j.immuni.2010.07.018 20727790 PMC3155777

[57] Mantovani A Cassatella MA Costantini C Jaillon S. Neutrophils in the activation and regulation of innate and adaptive immunity. Nat Rev Immunol (2011) 11:519–31. 10.1038/nri3024 21785456

[58] Phillipson M Heit B Colarusso P Liu L Ballantyne CM Kubes P. Intraluminal crawling of neutrophils to emigration sites: a molecularly distinct process from adhesion in the recruitment cascade. J Exp Med (2006) 203:2569–75. 10.1084/jem.20060925 17116736 PMC2118150

[59] Fazolini NPB Cruz ALS Werneck MBF Viola JPB Maya-Monteiro CM Bozza PT. Leptin activation of mTOR pathway in intestinal epithelial cell triggers lipid droplet formation, cytokine production and increased cell proliferation. Cell Cycle (2015) 14:2667–76. 10.1080/15384101.2015.1041684 26017929 PMC4614828

[60] Zhang T Tseng C Zhang Y Sirin O Corn PG Li-Ning-Tapia EM CXCL1 mediates obesity-associated adipose stromal cell trafficking and function in the tumour microenvironment. Nat Commun (2016) 7:11674. 10.1038/ncomms11674 27241286 PMC4895055

[61] Deladeriere A Gambardella L Pan D Anderson KE Hawkins PT Stephens LR The regulatory subunits of PI3K g control distinct neutrophil responses. Sci Signal (2015) 8:1–12. 10.1126/scisignal.2005564 25605974

[62] Puri KD Doggett TA Huang C Douangpanya J Hayflick JS Turner M The role of endothelial PI3Kgamma activity in neutrophil trafficking. Blood (2005) 106:150–7. 10.1182/blood-2005-01-0023 15769890 PMC1895128

[63] Camps M Rückle T Ji H Ardissone V Rintelen F Shaw J Blockade of PI3Kgamma suppresses joint inflammation and damage in mouse models of rheumatoid arthritis. Nat Med (2005) 11(9):936–43. 10.1038/nm1284 16127437

[64] Montecucco F Bianchi G Bertolotto M Viviani G Dallegri F Ottonello L. Insulin primes human neutrophils for CCL3-induced migration: crucial role for JNK 1/2. Ann N Y Acad Sci (2006) 1090:399–407. 10.1196/annals.1378.043 17384284

[65] Montecucco F Steffens S Burger F Da Costa A Bianchi G Bertolotto M Tumor necrosis factor-alpha (TNF-alpha) induces integrin CD11b/CD18 (Mac-1) up-regulation and migration to the CC chemokine CCL3 (MIP-1alpha) on human neutrophils through defined signalling pathways. Cell Signal (2008) 20:557–68. 10.1016/j.cellsig.2007.11.008 18164590

[66] Fang Y Vilella-Bach M Bachmann R Flanigan A Chen J. Phosphatidic acid-mediated mitogenic activation of mTOR signaling. Science (2001) 294:1942–5. 10.1126/science.1066015 11729323

[67] Kristof AS Marks-Konczalik J Billings E Moss J Stimulation of signal transducer and activator of transcription-1 (STAT1)-dependent gene transcription by lipopolysaccharide and interferon-gamma is regulated by mammalian target of rapamycin. J Biol Chem (2003) 278:33637–44. 10.1074/jbc.M301053200 12807916

[68] Lim HK Choi YA Park W Lee T Ryu SH Kim SY Phosphatidic acid regulates systemic inflammatory responses by modulating the Akt-mammalian target of rapamycin-p70 S6 kinase 1 pathway. J Biol Chem (2003) 278:45117–27. 10.1074/jbc.M303789200 12960176

[69] Lindemann SW Yost CC Denis MM McIntyre TM Weyrich AS Zimmerman GA. Neutrophils alter the inflammatory milieu by signal-dependent translation of constitutive messenger RNAs. Proc Natl Acad Sci U S A (2004) 101:7076–81. 10.1073/pnas.0401901101 15118085 PMC406468

[70] Rummel C Inoue W Poole S Luheshi GN. Leptin regulates leukocyte recruitment into the brain following systemic LPS-induced inflammation. Mol Psychiatry (2010) 15(5):523–34. 10.1038/mp.2009.98 19773811

[71] Aguliar-Valles A Kim J Jung S Woodside B Luheshi GN. Role of brain transmigrating neutrophils in depression-like behavior during systemic infection. Mol Psychiatry (2014) 19(5):599–606. 10.1038/mp.2013.137 24126927

[72] Landgraf MA Silva RC Corrêa-Costa M Hiyane MI Carvalho MHC Landgraf RG Leptin downregulates LPS-induced lung injury: role of corticosterone and insulin. Cell Physiol Biochem (2014) 33:835–46. 10.1159/000358656 24685581

[73] Beyrau M Bodkin JV Nourshargh S. Neutrophil heterogeneity in health and disease: a revitalized avenue in inflammation and immunity. Open Biol (2012) 2:120134. 10.1098/rsob.120134 23226600 PMC3513838

