# Supplementary material for: Leptin Mediates In Vivo Neutrophil Migration: Involvement of Tumor Necrosis Factor-Alpha and CXCL1
Source: Front Immunol. 2018 Feb 6;9:111. doi: 10.3389/fimmu.2018.00111 (PMC5808117; doi:10.3389/fimmu.2018.00111)
Supplement: Supplementary file 1 [file Presentation_1.PDF]

Supplementary Material

# Leptin mediates in vivo neutrophil migration: involvement of TNFalpha and CXCL1

*Glaucia Souza-Almeida, Heloisa D'Ávila, Patricia E. Almeida, Tatiana Luna, Sally Liechocki, Barbara Walzog, Ingrid Hepper, Hugo Castro-Faria-Neto, Patricia T. Bozza, Christianne Bandeira-Melo and Clarissa M. Maya-Monteiro**

**corresponding author _clarissa@ioc.fiocruz.br; clarissamayam@gmail.com*

Supplemental Fig. 1

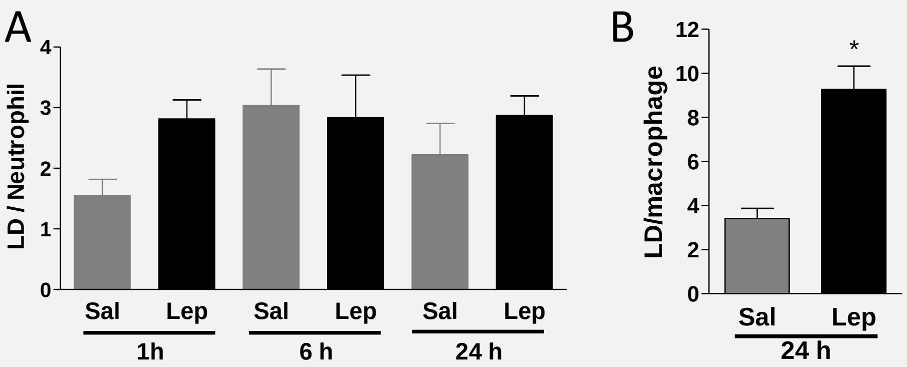

Fig. S1. Leptin did not induce lipid-bodies formation in peritoneal neutrophils. C57Bl/6 mice were injected intraperitoneally with leptin (1 mg/kg). Peritoneal cells were collected at 1h, 6h and 24 h after leptin injection and osmium tetroxide staining was performed as described (Maya-Monteiro et al., 2008). Graph shows lipid bodies count in peritoneal neutrophils (A) or macrophages (B). Each bar represents the mean ±SEM, minimum of n=3 and the experiment was performed 3 times. (A) Data were analyzed by Newman-Keuls-Student test and no statistically significant differences were observed. (B) Data was analyzed by analysis by Student t test. (*) Statistically significant difference ($p<0.05$) between leptin-stimulated and saline groups.

Supplemental Fig. 2

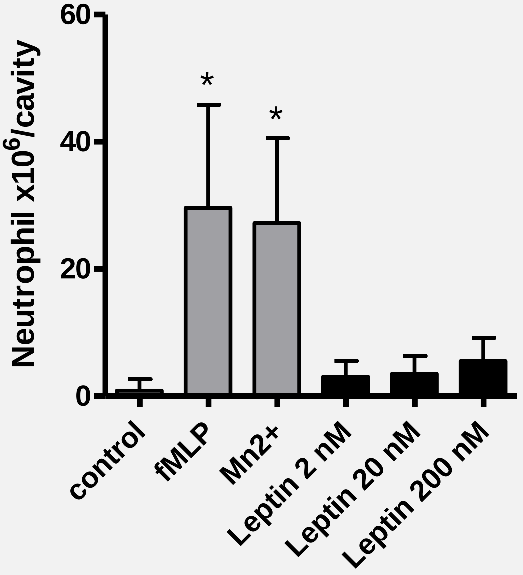

Fig. S2. Leptin does not directly induce neutrophil adhesion. Bone marrow neutrophils were isolated from naïve C57Bl/6 mice and exposed to immobilized fibrinogen upon stimulation with Mn^{2+} (3 mM) or fMLP (10 uM), or different leptin concentrations for 20 min. Each bar represents the mean ±SEM, n=3. Data were analyzed by Newman-Keuls-Student test. (*) Statistically significant differences ($p<0.05$) between stimulated and control groups.

Supplemental Fig. 3

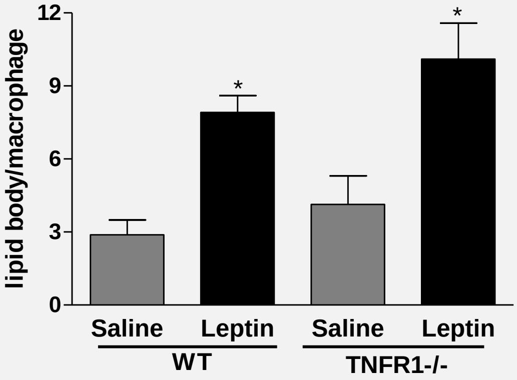

Fig. S3. Leptin induces lipid-bodies formation in peritoneal macrophages from TNFR1$^{-/-}$ mice. Leptin (1mg/kg) or saline was intraperitoneally injected in TNFR1$^{-/-}$ or TNFR1$^{+/+}$ mice and peritoneal washing was performed after 24h. Each bar represents the mean ±SEM, n=5-6. Data were analyzed by Newman-Keuls-Student test. (*) Statistically significant difference ($p<0.05$) between leptin and saline.
